# Effects of pegylated G-CSF on immune cell number and function in patients with gynecological malignancies

**DOI:** 10.1186/1479-5876-8-114

**Published:** 2010-11-09

**Authors:** Giuseppina Bonanno, Annabella Procoli, Andrea Mariotti, Maria Corallo, Alessandro Perillo, Silvio Danese, Raimondo De Cristofaro, Giovanni Scambia, Sergio Rutella

**Affiliations:** 1 Department of Gynecology and Obstetrics, Catholic University Med. School, Rome, Italy; 2 IRCCS in Gastroenterology, Istituto Clinico Humanitas, Milan, Italy; 3 Department of Medicine and Geriatrics, Hemostasis Research Centre, Catholic University Med. School, Rome, Italy; 4 Department of Hematology, Catholic University Med. School, Rome, Italy; 5 IRCCS San Raffaele Pisana, Rome, Italy

## Abstract

**Background:**

Pegylated granulocyte colony-stimulating factor (G-CSF; pegfilgrastim) is a longer-acting form of G-CSF, whose effects on dendritic cell (DC) and regulatory T cell (Treg) mobilization, and on the *in vivo *and ex vivo release of immune modulating cytokines remain unexplored.

**Methods:**

Twelve patients with gynecological cancers received carboplatin/paclitaxel chemotherapy and single-dose pegfilgrastim as prophylaxis of febrile neutropenia. Peripheral blood was collected prior to pegfilgrastim administration (day 0) and on days +7, +11 and +21, to quantify immunoregulatory cytokines and to assess type 1 DC (DC1), type 2 DC (DC2) and Treg cell mobilization. *In vitro*-differentiated, monocyte-derived DC were used to investigate endocytic activity, expression of DC maturation antigens and ability to activate allogeneic T-cell proliferation.

**Results:**

Pegfilgrastim increased the frequency of circulating DC1 and DC2 precursors. In contrast, CD4^+^FoxP3^+ ^*bona fide *Treg cells were unchanged compared with baseline. Serum levels of hepatocyte growth factor and interleukin (IL)-12p40, but not transforming growth factor-β1 or immune suppressive kynurenines, significantly increased after pegfilgrastim administration. Interestingly, pegfilgrastim fostered  *in vitro* monocytic secretion of IL-12p40 and IL-12p70 when compared with unconjugated G-CSF. Finally, DC populations differentiated *in vitro *after clinical provision of pegfilgrastim were phenotypically mature, possessed low endocytic activity, and incited a robust T-cell proliferative response.

**Conclusions:**

Pegfilgrastim induced significant changes in immune cell number and function. The enhancement of monocytic IL-12 secretion portends favorable implications for pegfilgrastim administration to patients with cancer, a clinical context where the induction of immune deviation would be highly undesirable.

## Background

Granulocyte colony-stimulating factor (G-CSF) can be administered to healthy individuals donating hematopoietic stem cells (HSC) for transplantation and to cancer patients with the aim to prevent and/or treat chemotherapy-induced neutropenia. Currently, primary prophylaxis with G-CSF is recommended in patients at high risk for febrile neutropenia based on age, medical history, disease characteristics and myelotoxicity of the chemotherapy regimen.

Filgrastim is a recombinant human G-CSF derived from *Escherichia coli*. Filgrastim has a short elimination half-life and requires daily subcutaneous injections for each chemotherapy cycle. The inconvenience associated with filgrastim administration has prompted the development of its covalent conjugation with monomethoxypolyethylene glycol (PEG) to obtain a longer-acting form (pegfilgrastim). The covalent attachment of PEG to the N-terminal amine group of the parent molecule increases its size, so that neutrophil-mediated clearance predominates over renal clearance in elimination of the drug, extending the median serum half-life of pegfilgrastim to 42 hours, compared with 3.5-3.8 hours for filgrastim [1]. However, the half-life is variable, depending on the absolute neutrophil count (ANC), which in turn reflects the ability of pegfilgrastim to sustain neutrophil production. The PEG group in the pegfilgrastim molecule is a relatively inert adduct and is expected not to alter granulocyte function significantly compared with filgrastim. In line with this assumption, pegfilgrastim retains the same biological activity as filgrastim, and binds to the same G-CSF receptor, stimulating neutrophil proliferation, differentiation and activation.

The long-term effects of long-acting growth factors such as pegfilgrastim are unknown. Because an increasing number of healthy donors and cancer patients are exposed to pharmacologic doses of G-CSF, a thorough understanding of G-CSF effects is imperative to safeguard donor and patient safety. In this respect, there is accumulating evidence that the biological activities of G-CSF are not limited to the myeloid lineage but extend to cell types and cytokine networks implicated in inflammation, immunity and angiogenesis [2]. Initial studies in mice supported a role for G-CSF in immune deviation towards T helper type 2 (Th2) cytokine production [3]. In humans, G-CSF increases interleukin (IL)-4 release and decreases interferon (IFN)-γ production [4], induces immune modulatory genes in T cells, including the Th2 master transcription factor GATA-3 [5], and promotes the differentiation of type 1 regulatory T cells (Treg), endowed with the ability to release IL-10 and transforming growth factor (TGF)-β1, and to suppress T-cell proliferation in a cytokine-dependent manner [6]. Furthermore, G-CSF induces the release of hepatocyte growth factor (HGF) [7], a pleiotropic cytokine that inhibits dendritic cell (DC) maturation [8] and down-regulates immune responses *in vivo *[9]. Finally, G-CSF mobilizes human type 2 DC (DC2) [10] and promotes the *in vitro *differentiation of regulatory DC through the stimulation of IL-10 and IFN-α production [11]. On a molecular level, G-CSF may determine mitochondrial dysfunction and proliferation arrest in T cells [12]. G-CSF-mobilized monocytes acquire the ability to release large quantities of immunosuppressive IL-10 and impair the induction of CD28-responsive complex in CD4^+ ^T cells [13]. Similar to filgrastim, pegylated G-CSF enhances the lipopolysaccharide (LPS)-stimulated production of immune suppressive IL-10 and favorably affects the clinical course of graft-versus-host disease (GVHD) in mice [14].

It is presently unknown whether pegylated G-CSF modulates human T-cell and DC function to a similar extent as unconjugated G-CSF. The hypothesis that the two formulations of G-CSF may target distinct cell populations *in vivo *and that, in spite of structural similarities, the spectrum of their biological activities may diverge is supported by investigations with human pegfilgrastim-mobilized HSC, which display unique features compared with HSC mobilized by filgrastim [15]. The present study provides evidence that pegylated G-CSF mobilizes both DC1 and DC2 precursors and, at variance with filgrastim, promotes monocytic IL-12 release. These findings portend favorable implications for pegfilgrastim administration to cancer patients.

## Methods

### Patient eligibility and treatment plan

The study population was comprised of 12 patients with gynecological malignancies (7 ovarian, 4 endometrial, 1 cervical cancer) ranging in age from 38 to 78 years (median age = 68 years). All patients received a conventional chemotherapeutic regimen, consisting of carboplatin (AUC5) and paclitaxel (175 mg/square meter). The patients' clinical characteristics are summarized in Table 1. After the completion of chemotherapy, patients were given a single dose (6 mg) of subcutaneous pegfilgrastim (Neulasta^®^; Amgen Dompè, Milan, Italy), as prophylaxis of febrile neutropenia. The investigations were approved by the Institutional Review Board. A retrospective analysis of 7 registrational clinical trials that examined the safety and efficacy of pegfilgrastim indicated that serum pegfilgrastim concentrations are consistently sub-therapeutic (< 2 ng/ml) by day +12 from the commencement of treatment [16]. Taking advantage of this knowledge, we collected blood samples from each consented patient on day 0 (the day before chemotherapy), and on days +7, +11 and +21.

**Table 1:** Patients' characteristics

Patient	Tumor (histotype)	FIGO Stage	Tumor grade	Number of previous chemotherapy cycles
UPN #1	Endometrial carcinoma (endometrioid)	Ic	G3	4

UPN #2	Endometrial carcinoma (serous)	IV	G3	5

UPN #3	Ovarian carcinoma (serous)	IIIb	G3	4

UPN #4	Cervical carcinoma (squamous)	Ib2	G2	2

UPN #5	Ovarian carcinoma (serous)	IIIc	G3	3

UPN #6	Endometrial carcinoma (mixed)	Ic	G2	1

UPN #7	Ovarian carcinoma (serous)	Ic	G3	4

UPN #8	Ovarian carcinoma	IIIc	G3	4

UPN #9	Ovarian carcinoma (serous)	IIIc	G3	4

UPN #10	Endometrial carcinoma (endometrioid)	Ic	G3	4

UPN #11	Ovarian carcinoma (endometrioid)	IIIc	G3	3

UPN #12	Ovarian carcinoma (endometrioid)	IIIb	G2	4

The demographic characteristics of the 12 patients enrolled in this study are shown. Patients had not received any chemotherapy in the 21 days preceding the commencement of the carboplatin/paclitaxel regimen (see Materials and Methods for further details). FIGO = International Federation of Gynecology and Obstetrics. UPN = Unique Patient Number.

A control group of 7 patients with gynecological malignancies received the same carboplatin/paclitaxel chemotherapy regimen, followed by daily filgrastim (5 μg/kg of body weight) from day +2 to day +10. Blood samples for *ex vivo *studies were drawn on day 0 (the day before chemotherapy) and on days +7, +11 (24 hours after the last filgrastim administration) and +21. For both groups of patients, serum was obtained by centrifugation at 4,000 rpm for 15 minutes shortly after blood collection, was divided into aliquots and stored at -80°C until used. Peripheral blood mononuclear cells (PBMC) were separated by Ficoll-Hypaque density gradient centrifugation, as previously reported [11], and were used as detailed below.

### Generation of monocyte-derived DC (Mo-DC) and evaluation of DC endocytic activity

CD14^+ ^monocytes were purified by negative selection (Monocyte Isolation Kit II, Miltenyi Biotec, Bergisch Gladbach, Germany) and were cultured in RPMI-1640 medium for 6 days at 37°C under serum-free conditions (10% BIT-9500; StemCell Technologies, Vancouver, BC) but in the presence of 500 IU/ml recombinant human GM-CSF and 25 ng/ml IL-4 (both cytokines were from R&D Systems, Oxon, Cambridge, UK). When indicated, the DC preparations were matured with 500 IU/ml tumour necrosis factor-α (TNF-α; R&D Systems) for 48 hours. Patient serum obtained before (pre-G) or after G-CSF administration (post-G) was supplemented to freshly isolated monocytes at 20% (v/v). In selected experiments, monocytes were stimulated *in vitro *with LPS (1 μg/ml) for 24 hours, prior to the measurement of secreted IL-12p40/IL-12p70 and IL-10 by ELISA.

To evaluate DC endocytic activity [17], monocyte-derived DC populations were suspended in culture medium supplemented with 10% fetal calf serum (FCS) in the presence of 100 μg/ml FITC-dextran (Sigma Chemical Co., St. Louis, MO) for 1 hour at 37°C. Control DC cultures were pulsed with FITC-dextran at 4°C, as previously detailed [8]. The extent of FITC-dextran incorporation was expressed as the ratio between the mean fluorescence intensity (MFI) of samples kept at 37°C and the MFI of samples cultured at 4°C, as detailed in the Figure legends.

### T-cell isolation and primary MLR

CD4^+ ^T cells were isolated from the peripheral blood with an indirect magnetic labeling system (CD4^+ ^T Cell Isolation Kit II; Miltenyi Biotec). Briefly, PBMC were labeled with a cocktail of biotin-conjugated antibodies against CD8, CD14, CD16, CD19, CD36, CD56, CD123, TCR γ/δ and CD235a. Anti-biotin microbeads were used for depletion, yielding a population of highly pure, untouched CD4^+ ^T cells. CD25 microbeads II (Miltenyi Biotec) were subsequently used for positive selection or depletion of CD25^+ ^cells, following the manufacturer's instructions.

CD4^+^CD25^- ^T cells were re-suspended in RPMI-1640 containing carboxyfluorescein-diacetate succinimidyl-ester (CFDA-SE, 2.5 μM; Molecular Probes, Eugene, OR) for 10 minutes at 37°C. To quench the labeling process, an equal volume of FCS was added. After washings in RPMI-1640 medium supplemented with 10% FCS, CD4^+^CD25^- ^T cells were activated with the mixed leukocyte reaction (MLR), as reported elsewhere [6]. Briefly, 5 × 10^4 ^allogeneic CD4^+^CD25^- ^T cells were cultured with fixed numbers of irradiated (25 Gy) DC or monocytes for 7 days, in RPMI-1640 medium supplemented with 20% BIT serum substitute. In selected experiments, serum from patients given either pegfilgrastim or filgrastim was supplemented at 20% (v/v) to the allogeneic MLR containing T cells and monocytes/DC from third-party healthy donors, as previously detailed [18].

### Immunological markers, four-color flow cytometry and data analysis

Mo-DC and monocytes were incubated for 20 minutes at 4°C with the following FITC-, PE-, PerCP- or PE-Cy7-conjugated monoclonal antibodies (mAb): CD1a, CD11c, CD14, CD80, CD86, CD83 (Caltag Laboratories, Burlingame, CA), HLA-DR, CD11c and IL-3 receptor α-chain or CD123 (BD Biosciences, Mountain View, CA), immunoglobulin-like transcript 3 (ILT3), DC-SIGN (DC-specific ICAM-3 grabbing non-integrin; CD209; Immunotech, Marseille, France), or with the appropriate fluorochrome-conjugated, isotype-matched irrelevant mAb to establish background fluorescence.

To monitor DC mobilization, peripheral blood samples were stained with a cocktail of FITC-conjugated mAb directed against lineage-specific antigens (CD4, CD14, CD16, CD19, CD20, CD56; Lineage Cocktail 1, BD Biosciences), and with anti-CD123, anti-HLA-DR and anti-CD11c mAb (BD), in order to discriminate type 1 DC (DC1) from DC2. Cells were then incubated with ammonium chloride lysis buffer for 5 minutes to remove residual red blood cells. Unfractionated whole blood samples were gated on the basis of forward and side scatter characteristics. After gating on lineage^-^HLA-DR^+ ^events, two populations of DC were identified, corresponding to HLA-DR^+^CD11c^+ ^DC (DC1) and HLA-DR^+^CD123^+ ^DC (DC2), as previously published [10]. The proportion of DC1 and DC2 within lineage^-/dim ^cells was enumerated and expressed as a percentage of total leukocytes.

The analysis of CFDA-SE fluorescence in cell proliferation tracking assays was performed with the proliferation wizard of the ModFit™ LT 2.0 software (Verity Software House Inc., Topsham, ME). Replication data were expressed in terms of proliferation index (PI), which was calculated as previously detailed [12].

The frequency of CD4^+^FoxP3^+ ^Treg cells in the peripheral blood of G-CSF-treated patients and in MLR cultures was estimated with an anti-FoxP3 mAb (PCH101 clone; eBioscience, San Diego, CA). Cells were initially stained with fluorochrome-conjugated anti-CD4 and anti-CD25 mAb (BD Biosciences), followed by sequential cell fixation and permeabilization and by labeling with the Alexa-Fluor^® ^488-conjugated anti-human FoxP3 mAb.

All samples were run through a FACS Canto^® ^flow cytometer (BD Biosciences) with standard equipment.

### Analysis of cytokine production

IL-12p40, IL-12p70, IL-10, TGF-β1 and HGF levels in patient serum and in culture supernatants were quantified by ELISA, using commercially available reagents (R&D Systems). The limits of detection were < 15 pg/ml IL-12p40, 0.625 pg/ml IL-12p70, 7.8 pg/ml IL-10, 7 pg/ml TGF-β1 and <40 pg/ml HGF.

### HPLC measurement of tryptophan (Trp) and kynurenine (Kyn)

Quantification of serum Trp and Kyn was obtained using reverse-phase (RP)-HPLC. The chromatographic procedure was similar to a method previously described, with minor modifications [19]. In brief, sample aliquots (100 μL) were deproteinized with HClO_4 _(0.3 M final concentration). After centrifugation (14,000 rpm for 15 minutes), the supernatants were spiked with 50 μM 3-L-nitrotyrosine and analyzed using a ReproSil-Pur C18-AQ (4 × 250 mm, 5 μM granulometry) RP-HPLC column (Dr. Maisch GmbH, Ammerbuch-Entringen, Germany), using a double-pump HPLC apparatus from Jasco (Tokyo, Japan) equipped with a mod. 2070 UV spectrophotometric detector and a FP-2020 fluorescence detector. Both detectors were connected in series to allow simultaneous measurements. The chromatographic peaks were detected by recording UV absorbance at 360 nm and emission fluorescence at 366 nm, after excitation at 286 nm. The elution solvent was: 2.7% CH_3_CN in 15 mM acetate buffer, pH 4.00 (both HPLC-grade from Fluka, Milan, Italy). To control the set-up and for peak quantification, Borwin 1.5 and MS Excel software were used. The concentrations of components were calculated according to peak heights and were compared both with 3-nitro-L-tyrosine as the internal standard and with the reference curves constructed with Kyn and L-Trp, both purchased from Sigma-Aldrich.

### Statistical analysis

The approximation of data distribution to normality was tested preliminarily using statistics for kurtosis and symmetry. Data were presented as median and interquartile range, and comparisons were performed with the Mann-Whitney test for paired or unpaired data, or with the Kruskal-Wallis test with Dunn's correction for multiple comparisons, as appropriate. The criterion for statistical significance was defined as *p *≤ 0.05.

## Results

### Effects of pegylated G-CSF on leukocyte subsets

**Figure 1:**
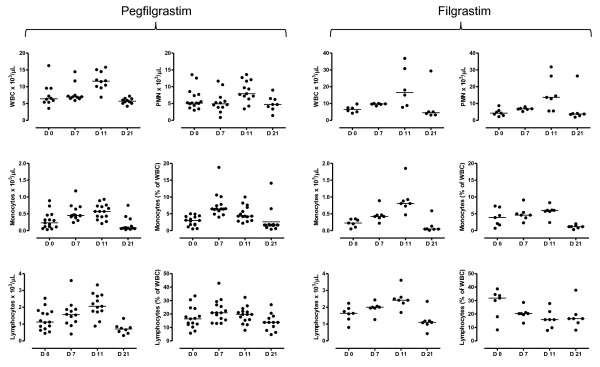
**Changes in leukocyte subsets in patients receiving growth factor support**. Leukocytes, neutrophils, monocytes and lymphocytes were enumerated with automated hematology analyzers before chemotherapy (day 0) and on days +7, +11 and +21 from G-CSF administration. Bars depict median values. The results of statistical comparisons among baseline and post-treatment samples and between the two study groups have been detailed in the main text.

Patients were initially evaluated for their white blood cell (WBC) and absolute neutrophil count (ANC) in response to pegfilgrastim. As depicted in Figure 1, both the WBC count and the ANC significantly increased on day +11 compared with pre-treatment values (*p *= 0.0002 and *p *= 0.033, respectively) and returned to baseline on day +21. Notably, filgrastim promoted a greater increase of WBC and neutrophils compared with pegfilgrastim, peaking on day +11 after the commencement of cytokine treatment (*p *= 0.0085 and *p *= 0.028 compared with baseline, respectively). Specifically, a median of 16.5 × 10^3 ^WBC/μl of blood (range 7.74-36.82) were counted in day +11 samples from filgrastim-treated patients compared with 11.64 × 10^3 ^WBC/μl of blood (range 6.88-15.78) in patients given pegfilgrastim (*p *< 0.05). Similarly, the ANC was significantly higher on day +11 after filgrastim administration (13.6 × 10^3^/μl, range 5.54-31.81) compared with the pegfilgrastim group (7.91 × 10^3^/μl, range 3.39-13.6; *p *< 0.05).

It has been previously shown that unconjugated G-CSF increases the number of lymphoid progenitors, mature lymphocytes and monocytes when administered to healthy HSC donors [20]. In our cohort of cancer patients, both pegfilgrastim and filgrastim significantly enhanced lymphocyte (*p *= 0.0002 and *p *= 0.0093, respectively) and monocyte counts (*p *< 0.0001 and *p *= 0.013, respectively) compared with baseline, peaking on day +11 from the commencement of cytokine treatment (Figure 1). Again, monocyte counts were significantly higher in patients treated with daily filgrastim (0.8 × 10^3 ^cells/μl, range 0.47-1.85, on day +11) compared with patients given pegfilgrastim (0.57 × 10^3 ^cells/μl, range 0.21-0.93; *p *= 0.04). Neither lymphocyte nor monocyte count at baseline differed significantly in the two patient cohorts (lymphocyte count = 1.69 × 10^3 ^cells/μl, range 0.8-2.24; and 1.21 × 10^3 ^cells/μl, range 0.45-2.54, in the filgrastim and pegfilgrastim group, respectively; monocyte count = 0.25 × 10^3 ^cells/μl, range 0.05-0.35; and 0.23 ± 0.06 × 10^3 ^cells/μl, range 0.03-0.89, in the filgrastim and pegfilgrastim group, respectively), suggesting that the sharper elevation of monocyte counts likely reflected an intrinsic ability of filgrastim to mobilize cells of the monocytic lineage. The observed changes in leukocyte subsets were transient, as indicated by the recovery of pre-treatment values by day +21 (Figure 1). Importantly, both the absolute number and the frequency of lymphocytes and monocytes increased as a result of pegfilgrastim administration (Figure 1), indicating the occurrence of mobilization and/or recruitment from peripheral sites into the circulation. However, the relative distribution of CD4^+ ^T cells, CD8^+ ^T cells, CD19^+ ^B cells and NK cells (defined as CD3^-^CD16^+^CD56^+ ^cells) within the lymphocyte population was unaffected by pegfilgrastim administration (data not shown). In sharp contrast to pegfilgrastim, filgrastim was unable to affect the frequency of lymphocytic and monocytic cells, as shown in Figure 1. The percentage of lymphocytes within total leukocytes was even lower on days +7 and +11 after filgrastim administration compared with baseline. Not unexpectedly, treatment with pegfilgrastim was associated with the mobilization of CD34-expressing HSC, which peaked on day +11 from cytokine treatment (4.2 cells/μl, range 2-23.1, compared with 0.9 cells/μl, range 0.5-10.4, at baseline; *p *< 0.05) and declined to pre-treatment values by day +21 (0.8 cells/μl, range 0.25-2).

### Mobilization of DC subsets and Treg cells

**Figure 2:**
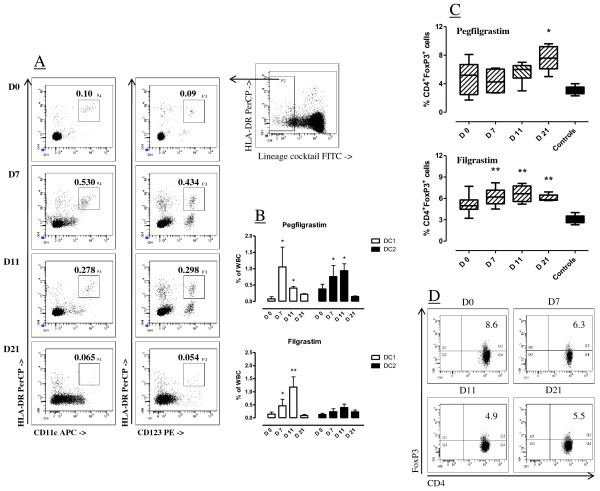
**Mobilization of DC precursors and Treg cells in patients receiving growth factor support**. The frequency of DC1 (lineage^-^HLA-DR^+^CD11c^+^) and DC2 (lineage^-^HLA-DR^+^CD123^+^) precursors and that of CD4^+^FoxP3^+ ^Treg cells was estimated by flow cytometry, as detailed in Materials and Methods. **Panel A**: Gating strategy for the enumeration of DC1 and DC2 precursors. Cells were initially gated based on lack of surface antigens associated with blood cell lineages. The co-expression of HLA-DR and CD11c or CD123 is shown in one patient given pegfilgrastim, and is representative of 12 independent experiments. **Panel B**: Cumulative frequency of DC1 (empty bars) and DC2 (black bars) in patients given pegfilgrastim or filgrastim. Median values and interquartile range are shown. *p < 0.05 compared with baseline. **p < 0.01 compared with baseline. **Panel C**: Boxes and whiskers depicting median values and interquartile range. *p = 0.01 compared with healthy controls (black bar); **p = 0.0009 compared with healthy controls (black bar). The Kruskal-Wallis test with Dunn's correction for multiple comparisons was used for statistical analyses. **Panel D**: Representative flow cytometry profile from one patient treated with pegfilgrastim. Quadrants were set according to the proper isotypic control (not shown). The percentage of CD4^+^FoxP3^+ ^T cells in indicated.

We next investigated whether pegfilgrastim induced changes in the frequency of circulating DC precursors. Cells were initially gated based on lack of expression of surface antigens associated with lineage differentiation, as detailed in Materials and Methods. A representative flow cytometry profile is shown in Figure 2A. Lineage^- ^cells were then analyzed for their expression of HLA-DR in association with CD11c (DC1) or CD123 (DC2), recognizing the IL-3 receptor α chain. Figure 2B depicts the cumulative frequency of DC1 and DC2 cells within the total leukocyte population of patients treated with either pegfilgrastim or daily filgrastim. In both cohorts of patients, cytokine administration translated into increased percentages of DC1 and DC2 cells, albeit with a different kinetics. Specifically, DC1 precursor cells were detected at higher frequency on day +7 after the commencement of pegfilgrastim (*p *< 0.05) and declined thereafter, whereas DC2 precursor cells reached a peak value on day +11 (*p *< 0.05). In contrast, daily filgrastim preferentially mobilized DC1 compared with DC2 cells, and both DC populations peaked at day +11 (*p *< 0.01 and *p *< 0.05 for DC1 and DC2, respectively), corresponding to the day after drug discontinuation (Figure 2B).

Because FoxP3^+ ^Treg cells are heterogeneous in humans and FoxP3-expressing cells have been detected both within CD4^+^CD25^+ ^and within CD4^+^CD25^- ^T-cell populations [21], we measured the frequency of *bona fide *Treg cells based on their CD4^+^FoxP3^+ ^phenotype. Treg cells at baseline were comparable in patients given pegfilgrastim (5.2%, range 1.7-8.1) and in patients treated with daily unconjugated G-CSF (4.9%, range 3.2-7.7), and significantly exceeded those in healthy volunteers (2.9%, range 2.3-4; nr of subjects = 8; *p *< 0.01), in agreement with other reports describing Treg expansion in the immunosuppressive *milieu *of patients with gynecological malignancies [22]. As shown in Figure 2C, a trend towards higher percentages of Treg cells was documented in samples collected after either pegfilgrastim or filgrastim administration. In the pegfilgrastim group, a median of 7.6% (range 5-9.6) CD4^+ ^T cells co-expressed FoxP3 on day +21 from cytokine administration compared with 5.2% (range 1.7-8.1) at baseline, but this difference failed to achieve statistical significance. Similarly, 5.8% (range 5.7-6.9) CD4^+^FoxP3^+ ^T cells were detected at late time-points after filgrastim administration compared with 4.97% (range 3.2-7.7) at baseline (*p *= NS). Notably, the percentage of Treg cells at any time-point after filgrastim treatment significantly exceeded that measured in healthy controls (Figure 2C). A representative experiment aimed at detecting Treg cells for one patient given pegfilgrastim is depicted in Figure 2D.

### Cytokine measurements and Trp/Kyn ratio

**Figure 3:**
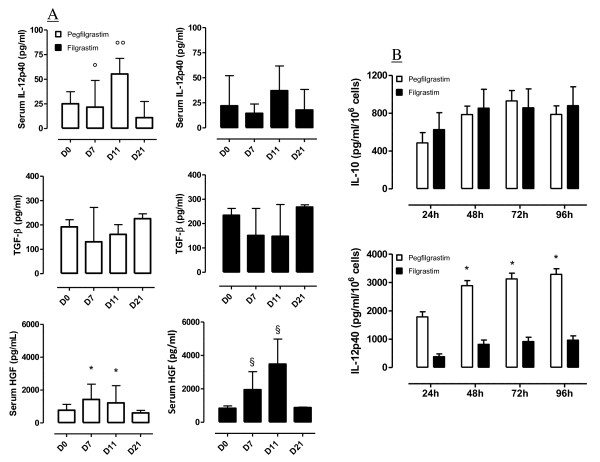
***Ex vivo *cytokine measurements and *in vitro *monocytic release of IL-10 and IL-12p40**. **Panel A**: Patient serum was collected at the indicated time-points and used to evaluate IL-12p40, TGF-β1 and HGF levels by ELISA. Bars depict median values and interquartile ranges recorded in 12 independent experiments performed in duplicate. °*p *< 0.01 when comparing IL-12p40 levels on day +7 vs. day +21. °°*p *= 0.0036 when comparing IL-12p40 levels on day +11 vs. baseline and vs. day +21. *p = 0.0023 when comparing HGF levels on day +7 and day +11 vs. baseline. §*p *= 0.0062 when comparing HGF levels on day +7 and day +11 vs. baseline and vs. day +21. **Panel B**: Monocytes were purified on day +11 from the commencement of cytokine treatment, coincident with maximal mobilization into the peripheral blood. Cells (1 × 10^6^) were stimulated with 1 μg/ml LPS in complete culture medium for up to 96 hours. Supernatants were harvested daily and used to measure IL-10 and IL-12p40 by ELISA. IL-10 and IL-12p40 levels were also estimated in 7 patients with gynecological cancers treated with daily G-CSF. Median values and interquartile range are shown. *p < 0.01 compared with IL-12p40 levels in supernatants of post-filgrastim monocytes.

It is now recognized that the balance between IL-12 and IL-10 produced by the antigen presenting cell compartment dictates the outcome of an immune response, with IL-12 release leading to robust T-cell priming and IL-10 secretion primarily mediating the induction of T-cell unresponsiveness [23]. As shown in Figure 3A, serum IL-12p40 levels significantly increased after pegfilgrastim administration and returned to baseline on day +21. Conversely, IL-12p40 slightly declined in cancer patients given daily G-CSF, and returned to pre-treatment values by day +11. IL-10 serum levels were consistently below the ELISA lowest standard (7.8 pg/ml), either in patients treated with pegfilgrastim or in those given unconjugated G-CSF (data not shown). TGF-β and HGF play significant roles as immune modulating growth factors both physiologically and in pathological states such as cancer. In order to gain further insights into the immune modulation exerted by G-CSF, we also measured TGF-β and HGF levels before and after cytokine treatment. TGF-β levels displayed minor fluctuations in the peripheral blood of patients given either unconjugated G-CSF or pegylated G-CSF (Figure 3A). In contrast, the administration of pegfilgrastim was associated with an increase of serum HGF compared with baseline (Figure 3A). Importantly, serum HGF levels on day +11 were significantly higher in patients receiving filgrastim than in those given pegfilgrastim (*p *= 0.043). In both cohorts of patients, HGF returned to pre-treatment values on day +21 from the commencement of cytokine administration.

Because HGF may induce the expression of indoleamine 2,3-dioxygenase 1 (IDO1) [8], an enzyme implicated in the conversion of Trp into immune suppressive Kyn [24], we analyzed IDO1 mRNA expression in patient monocytes and neutrophils and measured serum Trp and Kyn levels after treatment with pegfilgrastim. RT-PCR studies with purified monocytes and neutrophils indicated that mRNA signals for IDO1 were unchanged after pegfilgrastim administration [**see **Additional file 1]. As shown in Additional file 1, serum Kyn displayed minor fluctuations following pegfilgrastim administration. It should be emphasized that Kyn levels in 4 out of 5 patients, either at baseline or after the clinical provision of pegfilgrastim, were higher than those measured in healthy controls. Finally, serum Trp levels were significantly lower (< 40 μM) than in healthy controls (83.9 μM on average; data not shown) at any time-point, in line with previous data on altered Trp catabolism in cancer patients [24].

**Figure 4:**
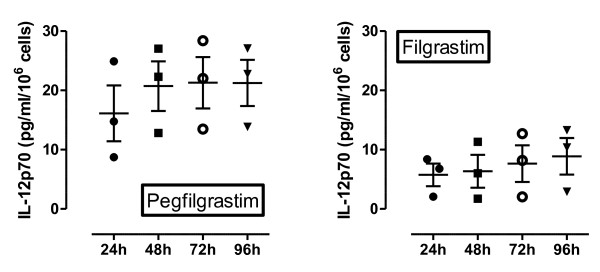
***In vitro *monocytic release of bioactive IL-12p70**. Monocytes (1 × 10^6^) purified from the peripheral blood of patients given pegfilgrastim (n = 3) or filgrastim (n = 3) were stimulated with LPS as detailed in the legend to Figure 3B. Supernatants were harvested daily and used to measure IL-12p70 by ELISA. Each point is representative of the mean value of triplicate IL-12p70 measurements.

In order to more accurately substantiate the assumption that pegfilgrastim alters the balance between IL-12 and IL-10, monocytes, a prominent cellular source of both IL-12 and IL-10, were magnetically purified on day +11 from the peripheral blood of patients treated with pegfilgrastim (24 hours before the anticipated decline of serum pegfilgrastim concentration [16] and coincident with maximal monocyte mobilization) and from cancer patients treated with daily filgrastim (24 hours after the last G-CSF administration). Monocytes were routinely > 95% pure, as evaluated by flow cytometry measurements of CD14 expression (data not shown). Equal numbers of monocytes from pre-G-CSF and post-G-CSF samples were cultured for up to 96 hours in the presence of LPS as a stimulus. The LPS-induced monocytic release of IL-10 increased after pegfilgrastim administration (Figure 3B). Notably, post-pegfilgrastim monocytes secreted considerable amounts of IL-12p40 at any time-point in culture (Figure 3B). In line with previous reports [25], monocytes from filgrastim-treated patients secreted low amounts of IL-12p40. Intriguingly, IL-12p40 production by post-filgrastim monocytes was significantly lower than that measured in post-pegfilgrastim monocyte cultures at any time-point. To further reinforce the assumption that pegfilgrastim, but not unconjugated G-CSF, enhances the monocytic release of IL-12 on a *per cell *basis, IL-12p70 levels were measured in supernatants of monocytes purified from 3 patients given pegfilgrastim and 3 patients receiving unconjugated G-CSF. As shown in Figure 4, post-pegfilgrastim monocytes released significantly higher levels of IL-12p70 compared with monocytes isolated from cancer patients treated with unconjugated G-CSF.

### In vitro DC phenotype and function

It has been previously shown that filgrastim indirectly affects DC number and function, skewing *in vitro *DC differentiation towards a tolerogenic profile [10,11]. To assess whether soluble factors induced by pegfilgrastim hindered DC maturation, we cultured monocytes from healthy controls with patient serum collected either before or after G-CSF administration. At the end of the 6-day culture period, cells were recovered and labeled with a panel of mAb recognizing DC activation/differentiation antigens. Control cultures consisted of immunogenic DC differentiated with GM-CSF and IL-4 under serum-free conditions. The phenotypic and functional features of the DC-like cells differentiated after the provision of filgrastim have been extensively reported elsewhere [11] and these experiments were not further replicated in the present study.

**Figure 5:**
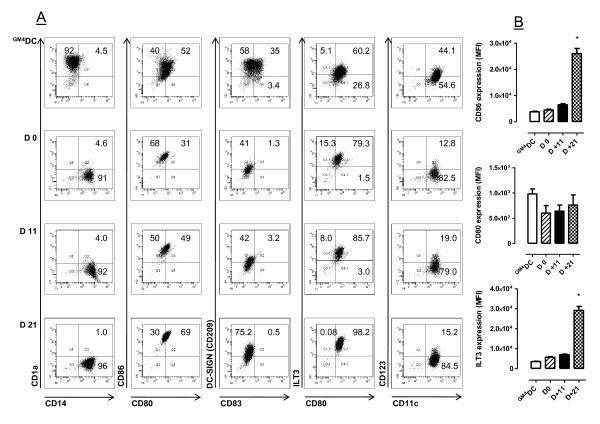
**Phenotypic features of DC-like cells from patients receiving pegfilgrastim**. Monocytes were purified from the peripheral blood of patients given pegfilgrastim and were cultured in the presence of either pre-G-CSF or post-G-CSF serum (20% v/v) for 6 days, as detailed in Materials and Methods. Control cultures consisted of immunogenic DC preparations that were differentiated with GM-CSF and IL-4 without the provision of additional maturation stimuli (^GM4^DC). **Panel A**: Percentage of cells staining positively for a given antigen in a representative experiment out of 12 with similar results. **Panel B**: Relative expression of informative differentiation antigens. Median values and interquartile range recorded in 12 independent experiments. *denotes a *p *value < 0.05 compared with the other time-points.

**Figure 6:**
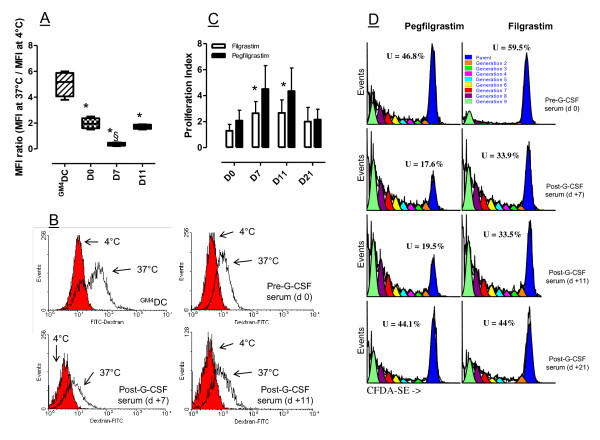
**Functional features of Mo-DC from patients receiving pegfilgrastim**. Monocytes were purified from the peripheral blood of patients given pegfilgrastim and were cultured in the presence of either pre-G-CSF or post-G-CSF serum (20% v/v) for 6 days, as detailed in Materials and Methods. Control cultures consisted of immunogenic DC preparations that were differentiated with GM-CSF and IL-4 without the provision of additional maturation stimuli (^GM4^DC). **Panel A**: Uptake of FITC-conjugated dextran by monocytes cultured *in vitro *in the presence of pre-pegfilgrastim serum (day 0) or post-pegfilgrastim serum (days +7 and +11). Median values and interquartile range are shown. *p < 0.05 compared with Mo-DC differentiated with GM-CSF and IL-4; §*p *< 0.05 compared with cells cultured with pre-G-CSF serum. **Panel B**: Representative experiment; red histograms depict the uptake of FITC-conjugated dextran by monocytes kept at 4°C (negative control) and empty histograms depict the uptake of FITC-conjugated dextran by the monocyte preparations kept at 37°C. **Panel C**: CD4^+^CD25^- ^T cells and monocytes were purified from the peripheral blood of healthy donors as detailed in the main text. After irradiation, monocytes were cultured with CFDA-SE loaded, allogeneic T cells at a fixed monocyte-to-T cell ratio (1:27) for 7 days, either in the absence or presence of patient serum (20% v/v). The proliferation index of T-cell cultures established in the presence of patient serum collected before and after G-CSF administration is shown. The bars depict median and interquartile range recorded in 3 independent experiments performed in duplicate. **Panel D**: Results of a representative experiment out of 3 with similar results. The percentage of parental, undivided cells (U; depicted in blue) is indicated. The analysis of CFDA-SE fluorescence was performed with the proliferation wizard of the ModFit software package, as previously detailed [12].

For technical reasons, insufficient quantities of day +7 serum were obtained to be supplemented at 20% v/v to the DC and monocyte cultures. Figure 5 thus illustrates a representative experiment with day +11 and day +21 monocyte-derived DC preparations. Not unexpectedly, monocytes cultured with GM-CSF and IL-4 under serum-free conditions down-regulated CD14, were uniformly CD1a^+^, and up-regulated costimulatory molecules (CD80 and CD86) and DC maturation antigens such as CD83 and CD209 (Figure 5A). In sharp contrast, monocytes cultured with either pre- or post-pegfilgrastim serum maintained a CD14^+^CD1a^- ^phenotype, in accordance with previous reports on the phenotype of human serum-supplemented DC cultures [11]. Interestingly, monocyte cultures containing pre- and post-pegfilgrastim serum differed in their expression of costimulatory molecules. CD80 and CD86 were expressed at significantly higher levels after culture with post-pegfilgrastim serum, both in terms of percent positive cells and in terms of MFI (Figure 5A and 5B). In addition, post-pegfilgrastim monocytes up-regulated the DC maturation antigen CD209 compared with cells in pre-G-CSF cultures (Figure 5B). ILT3 was also detected on higher percentages of post-pegfilgrastim monocytic cells, where its expression increased in terms of fluorescence intensity. Finally, CD83, CD11c and CD123 were detected on comparable percentages of pre-G-CSF and post-G-CSF monocytes. Taken together, phenotypic studies revealed that soluble factors contained in post-pegfilgrastim serum promoted the acquisition of a mature DC-like phenotype, with high expression of costimulatory molecules and CD209, and preserved expression of the monocyte/macrophage antigen CD14. In line with this, monocytes nurtured with post-pegfilgrastim serum possessed a diminished ability to endocytose FITC-conjugated dextran, a measure of DC maturation status, compared with monocytes cultured with pre-pegfilgrastim serum and with immature DC differentiated with GM-CSF and IL-4, used as control for optimal incorporation of FITC-dextran (Figure 6A and 6B).

### Effect of post-G-CSF serum on alloantigen-induced T-cell proliferation

We finally asked whether the DC-like preparations obtained after culture of monocytes from G-CSF-treated patients could differentially activate the proliferation of naïve allogeneic CD4^+^CD25^- ^T cells in comparison with conventional immunogenic DC differentiated with GM-CSF and IL-4. To this end, allogeneic naïve CD4^+^CD25^- ^T cells were pre-loaded with the fluorescent dye CFDA-SE and were then cultured with patient DC or monocytes at escalating ratios. As shown in Additional file 2, T-cell proliferation as detected by the progressive halving of CFDA-SE fluorescence was superimposable under the culture conditions here established, suggesting that the alloantigen-presenting capacity of *in vitro *differentiated DC-like cells was unaffected by the *in vivo *exposure to pegfilgrastim. In a further set of experiments, either pre- or post-pegfilgrastim serum were supplemented to allogeneic MLR cultures to assess whether soluble factors in post-pegfilgrastim serum regulate an ongoing T-cell response to monocytes from third-party healthy donors. As shown in Figure 6C, the provision of post-pegfilgrastim serum (days +7 and + 11) to an allogeneic MLR culture translated into higher levels of T-cell proliferation compared with cultures supplemented with post-filgrastim serum collected at the same time-points (Figure 6C and 6D). Modeling of CFDA-SE profiles reinforced the concept that higher percentages of undivided, parental cells were contained within MLR cultures supplemented with serum from patients receiving filgrastim [see Additional file 3], thus suggesting that pegfilgrastim-induced soluble factors were less likely to restrain T-cell proliferative responses *in vitro *than filgrastim-elicited immune suppressive mediators [18].

## Discussion

It is conceivable that the G-CSF formulations currently available for clinical use differentially affect WBC number and function. For instance, a direct comparison of lenograstim (nonglycosylated G-CSF) and filgrastim or pegfilgrastim with regard to neutrophil phenotype and function indicated that neutrophils primed with lenograstim are less functional and structurally more immature compared with those primed with filgrastim and, to a lesser extent, pegfilgrastim [26]. Importantly, randomized clinical trials evaluating single administration of pegfilgrastim vs. daily filgrastim as an adjunct to chemotherapy in patients with hematological and solid malignancies reported similar efficacy profiles [27] or even a lower overall rate of febrile neutropenia in patients treated with pegfilgrastim compared with those given daily filgrastim [28].

The present study aimed to address whether pegfilgrastim given as prophylaxis for chemotherapy-induced neutropenia affects the number and function of immune cells, a finding with potential implications for the treatment of cancer patients. The immune modulating actions of unconjugated G-CSF have been previously described both *in vitro *and *ex vivo *[29]. This basic knowledge has been translated into animal models of autoimmune disorders to skew the immune response and to promote tolerance. For instance, G-CSF ameliorated experimental autoimmune encephalomyelitis [30], type 1 diabetes [31], experimental colitis [32] and lupus nephritis [33] through effects on adaptive and innate immune responses. A pilot clinical trial in Crohn's disease provided *proof of principle *in favor of immune regulatory effects by filgrastim in the human setting [34]. In this study, daily treatment with G-CSF for 4 weeks was correlated with an increase of IL-10-secreting type 1 Treg cells in the peripheral blood and with the accumulation of plasmacytoid DC in the gut *lamina propria *[34].

In the present report, WBC and ANC recovery in patients treated with pegfilgrastim occurred without the fluctuations associated with daily filgrastim injections. The administration of pegfilgrastim translated into a transient but significant elevation of CD34-expressing HSC, lymphocytes and monocytes. Lymphocyte recirculation is expected to favorably impact on the immune control of the underlying malignancy, and the observation that prompt lymphocyte recovery predicts a higher relapse-free survival in cancer patients [35] underpins the potential clinical significance of the pegfilgrastim-induced changes in WBC subsets. Pegfilgrastim did not elicit any appreciable mobilization of Treg cells, as documented by serial measurements of the frequency of circulating CD4^+^FoxP3^+ ^Treg cells. We cannot rule out the possibility that any G-CSF-induced recirculation of Treg cells was obscured by the high frequency of Treg cells already measured at baseline. Of interest, filgrastim has been reported to increase the frequency of CD4^+^CD25^high ^Treg cells only when given to cancer patients in combination with cyclophosphamide as HSC mobilization regimen [36]. In healthy donors, the phenotype and frequency of CD4^+^CD25^high^FoxP3^+ ^Treg cells may be unaffected by G-CSF [37]. At variance with human data, filgrastim recruited functional TGF-β-expressing Treg cells to the pancreatic lymph nodes of NOD mice, with the likely aim to restrain the proliferation and function of diabetogenic T cells [31]. It remains to be determined whether Treg recirculation and/or recruitment to sites of inflammation and tissue injury may also occur in humans as a result of pegfilgrastim administration.

We were also interested in evaluating whether pegfilgrastim induced the release of immune suppressive HGF and TGF-β1. HGF is a pro-angiogenic and tumor-promoting cytokine. HGF reportedly skews DC function, driving an IL-10-secreting tolerogenic profile both in mice [38] and in humans [8]. We measured significantly elevated levels of HGF in patients treated with either pegfilgrastim or filgrastim. Furthermore, HGF secretion was significantly lower after pegfilgrastim compared with daily filgrastim administration. In contrast, serum TGF-β1 levels were not modified by either G-CSF formulation. The observation that HGF induces functional IDO1 in human monocyte-derived DC [8] raised the previously unexplored possibility that pegfilgrastim may indirectly activate IDO1-mediated Trp breakdown into immune suppressive derivatives, collectively referred to as Kyn. Interestingly, serum Kyn were not significantly different when comparing samples at baseline with those obtained from patients receiving pegfilgrastim. It should be noted that baseline Kyn levels in our patient cohort were higher than those measured in healthy controls (median Kyn concentration = 1.86 μM; number of samples = 20), probably reflecting the expression of functional IDO1 by the ovarian and endometrial cancer cells [39]. Also, mRNA signals for IDO1 in monocytes and granulocytes, a potential source of IDO1 activity [40], were unchanged when comparing pre-G and post-G samples. These observations are backed by a recent study indicating that G-CSF-mobilized immature myeloid cells inhibit alloreactive responses in mice through an IDO-independent mechanism, and that G-CSF signaling is incapable of directly inducing IDO [41].

The studies published so far suggest that the extent of DC1/DC2 mobilization by filgrastim crucially depends on the intensity of the mobilization regimen and on the underlying neoplastic disorder. In this respect, filgrastim preferentially mobilized DC2 in healthy donors [10] but failed to impact on the DC1/DC2 ratio in patients with hematological and solid malignancies [42]. In another study with healthy donors, low-dose G-CSF (8-10 μg/kg/day) increased the frequency of CD123^+ ^blood DC precursors but mobilized CD11c^+ ^DC only occasionally [43]. Furthermore, high-dose G-CSF (30 μg/kg/day) mobilized CD123^+ ^DC in patients with multiple myeloma but only occasionally in those affected by non-Hodgkin's lymphoma, and exerted varying effects on CD11c^+ ^DC [43]. We have shown herein that pegfilgrastim mobilized both DC1 and DC2 precursors into the peripheral blood of patients with gynecological malignancies treated with carboplatin and paclitaxel, suggesting lack of DC skewing *in vivo*. The highest frequencies of DC1 precursors were measured on day +7 from pegfilgrastim administration, whereas DC2 precursors were higher in day +11 samples and declined thereafter. It is conceivable that different chemotherapy/growth factor combinations and doses and/or intrinsic characteristics of the underlying neoplastic disorder account for differences in the relative proportion of DC1/DC2 precursors and in their mobilization kinetics. It is presently unknown whether the transient DC1 mobilization induced by pegfilgrastim will impact on the host immune system's ability to control disease progression.

IL-12, a prototype member of a family of IL-12-related cytokines that includes IL-23 and IL-27, is an instigator of Th1 immune responses and possesses *in vivo *anti-tumor activities [44]. IL-12 is a heterodimer formed by a 35-kDa light chain (known as p35 or IL-12α) and a 40-kDa heavy chain (known as p40 or IL-12β). Messenger RNA encoding IL-12p35 is present in many cell types, whereas mRNA encoding IL-12p40 is restricted to cells that produce the biologically active heterodimer [45]. DC and monocytes have been reported to secrete a 10-1,000 fold excess of IL-12p40 compared with IL-12p75 [44]. A report on post-transplantation immune functions in 43 patients receiving filgrastim has shown that cytokine administration delays the reconstitution of CD4^+ ^T cells and blunts anti-fungal T-cell responses [25]. These abnormalities were correlated with the inability of DC and monocytes from G-CSF-treated patients to release IL-12p40 [25]. Interestingly, the *in vivo *immune modulating effects of G-CSF were replicated *in vitro *when monocytes from normal volunteers were differentiated along the DC lineage after their 24-hour pre-treatment with exogenous G-CSF. Under these conditions, IL-12p40 production was inhibited both at the mRNA and protein level [25]. In our study, pegfilgrastim administration was associated with a significant increase of the inducible IL-12p40 subunit in patient serum. In patients given filgrastim, IL-12p40 slightly declined and returned to baseline values by day +11 from the commencement of cytokine treatment. Interestingly, neutrophil-derived serine proteases have been reported to inactivate human growth factors such as TNF-α at sites of inflammation and to promote the formation of cytokine split products [46]. It is tempting to speculate that immunoreactive IL-12 in patients given filgrastim may have been degraded as a result of sharp increases in circulating PMN capable of releasing proteolytic enzymes. Intriguingly, monocytes from patients treated with pegfilgrastim released higher amounts of both IL-12p40 and IL-12p70 *in vitro *compared with monocytes from filgrastim-treated patients. In contrast, the LPS-induced release of IL-10 increased to a similar extent in cultures established with monocytes from patients given pegfilgrastim and filgrastim. IL-12p40 homodimers may behave as IL-12 receptor antagonists both in mice and in humans, inhibiting IL-12-induced T-cell proliferation [47,48]. Our observation that post-pegfilgrastim monocytes release higher amounts of bioactive IL-12p70 compared with post-filgrastim monocytes supports the conclusion that pegfilgrastim may not dampen *in vivo *anti-tumor immunity and/or host defense against infectious agents, a response that crucially depends on the balance between IL-12 and IL-10 production. It has been reported that 6-sulfo LacNAc^+ ^DC, a major source of IL-12 and potent inducers of T-cell responses *in vitro*, are efficiently mobilized in healthy donors given G-CSF at 7.5 μg/kg of body weight [49]. Conceivably, pegfilgrastim might also favor the mobilization of 6-sulfo LacNAc^+ ^DC or other as yet unrecognized monocyte/DC populations with a unique ability to produce bioactive IL-12.

It is known that unconjugated G-CSF promotes the development of tolerogenic DC *in vitro *[11] and *in vivo *[31]. We showed herein that pegfilgrastim-induced soluble factors promoted the emergence of mature DC-like populations with high expression of costimulatory molecules (CD80, CD86), CD83 and CD209, and with low endocytic capacity. Post-pegfilgrastim DC-like cells also up-regulated ILT3, an inhibitory receptor detected on anergizing DC preparations [50,51], and yet activated the proliferation of allogeneic naïve T cells to a similar extent as immunogenic DC. It should be noted that ILT3 expression may be dispensable for the induction of CD4^+^CD25^+ ^Treg cells by 1,25-dihydroxyvitamin D3 [52], indicating that molecular determinants of T-cell suppression other than ILT3 may be operational depending upon the experimental system. Of potential interest, we measured high levels of IL-10 in post-pegfilgrastim DC cultures (317 ± 140 pg/ml compared with 27.1 ± 2.3 pg/ml in control cultures of immunogenic ^GM4^DC). IL-10 secretion may have been responsible for ILT3 up-regulation on post-pegfilgrastim monocytes, in line with the effect of exogenous IL-10 on ILT3 expression by human vascular endothelial cells [53]. We also evaluated the ability of post-pegfilgrastim DC to activate allogeneic T-cell responses *in vitro*. Interestingly, monocytes from patients given pegfilgrastim induced T-cell proliferation to a similar extent as immunogenic DC. In line with this, T-cell proliferation in response to allogeneic monocytes was not inhibited by the provision of post-pegfilgrastim serum to the MLR culture. Our observations on *in vitro *DC phenotype and function reinforce the view that pegfilgrastim and filgrastim differ in their ability to skew monocyte function, with the former supporting the *in vitro *development of activating DC and the latter favoring the emergence of tolerogenic DC preparations [18].

## Conclusions

Taken together, the experimental evidence herein presented indicates that the administration of pegfilgrastim to hasten neutrophil recovery should not translate into undesired immune suppression in cancer-bearing patients, who might benefit from robust monocytic production of IL-12, in the absence of excessive induction of immune suppressive IL-10 and HGF. A further implication of our findings pertains to HLA-matched stem cell transplantation, a clinical context where pegfilgrastim administration may modulate the number of immune cells and/or levels of immune regulatory soluble factors, thus ameliorating leukemia clearance. In this respect, it has been shown that multiple pegylation of G-CSF imparts an enhanced biological activity with respect to immune cells and improves stem cell transplant in mice [54]. Intriguingly, multi-pegylated versions of G-CSF separate GVHD from graft-versus-leukemia (GVL) through the activation of invariant NKT cells, thus contributing to leukemia eradication [55,56]. These considerations add to the knowledge that pegfilgrastim has advantages over filgrastim in terms of patient compliance, ease of administration and patient quality of life [1]. Whether the pegfilgrastim-induced modulation of immune function will favorably impact on disease control in cancer-bearing patients remains to be prospectively determined.

## Competing interests

The authors declare that they have no competing interests.

## Authors' contributions

GB carried out the experiments and participated in the design of the study. AM, AP and MC carried out the experiments. AP and GS participated in the design of the study and were responsible for patient care and sample procurement. RDC carried out the experiments and contributed to manuscript drafting. SD gave intellectual input and advice. SR participated in the design of the study, carried out the experiments, performed the statistical analysis and drafted the manuscript. All authors read and approved the final manuscript.

## Supplementary Material

Additional file 1 **Expression of IDO1 mRNA and serum Kyn levels in patients given pegfilgrastim**. **Panel A**: Expression of IDO1 mRNA in patient monocytes and granulocytes. Details on RNA extraction and reverse-transcription were previously published [8]. The following primers were used for mRNA amplification: 5'-ACTGCCCCTGTGATAAACTGTGG-3' and 5'-GCGTGTGCCATTCTTGTAGTCTG-3' (human IDO1; GI 156071492); 5'-TGACATCAAGAAGGTGGTGA-3' and 5'-TCCACCACCCTGTTGCTGTA-3' (human GAPDH; GI 7669491). Primer sets were designed using the Beacon Design Software (Version 3) and the sequences available in the Gene Bank™ database. All nucleotide primers were synthesized by MWG (Florence, Italy), and PCR products were analyzed on 3% agarose gel (Agarose, type XII: low viscosity for beading, Sigma Aldrich) stained with ethidium bromide. M = marker. + = normal endometrial tissue used as positive control for IDO1 mRNA expression. **Panel B**: Quantitative densitometry (Quantity One software; Bio-Rad, Hercules, CA) is shown with monocytes and granulocytes isolated from 2 patients given pegfilgrastim. Insufficient numbers of cells were available on day +7, and PCR analyses were performed with patient material obtained on days 0, +11 and +21. Normal endometrial tissue was used as positive control for IDO1 mRNA expression (red column). **Panel C**: Serum Kyn levels were measured by RP-HPLC in 5 patients before (day 0) and after pegfilgrastim administration (days +7, +11 and +21), as detailed in Materials and Methods. Data from each individual patient have been plotted using a different color. The dotted line indicates the median serum Kyn concentration measured in 50 healthy subjects (2.3 μM). Click here for file

Additional file 2 **T-cell stimulation by Mo-DC generated *in vitro *after *in vivo *administration of pegfilgrastim**. Mo-DC were differentiated from patient monocytes in the presence of either pre-G-CSF serum or post-G-CSF serum (collected on day + 11), as detailed in Materials and Methods. Immunogenic DC were generated with IL-4 and GM-CSF, in accordance with established DC differentiation protocols [17]. The Mo-DC preparations were co-cultured with CFDA-SE pre-loaded, allogeneic naïve CD4^+^CD25^- ^T cells at different T cell-to-DC ratio. The percentage of CD25-expressing, CFDA-SE^dim ^and CFDA-SE^bright ^cells is indicated. One representative experiment out of 5 with similar results is shown. Click here for file

Additional file 3 **Cell proliferation tracking after provision of post-G-CSF serum to MLR cultures**. MLR cultures were established as above detailed. T cells and monocytes were plated at a fixed DC-to-T cell ratio (1:27). The percentage of proliferating T cells residing within each cell generation (G) was calculated with the proliferation wizard of the ModFit™ software. Median values and interquartile range are shown. *denotes a *p *value < 0.05 when comparing the percentage of parental (P), undivided cells in MLR cultures established with serum from patients given pegfilgrastim (black bars) or filgrastim (empty bars). Click here for file
