# Supplementary figures and images for: Effects of pegylated G-CSF on immune cell number and function in patients with gynecological malignancies
Source: J Transl Med. 2010 Nov 9;8:114. doi: 10.1186/1479-5876-8-114 (PMC2992497; doi:10.1186/1479-5876-8-114)

## Slide 1
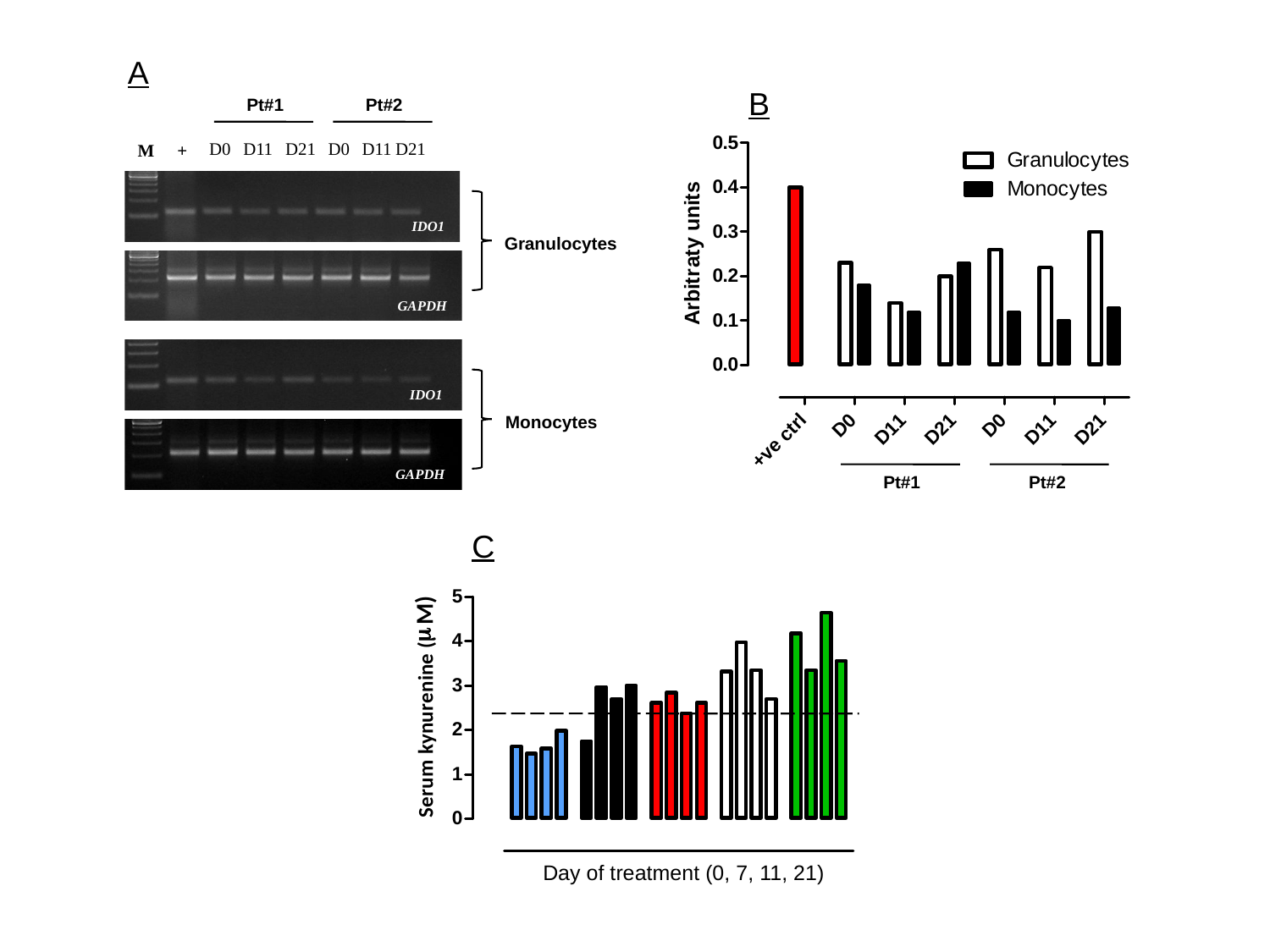

A
Pt#1
Pt#2
D0
D11
D21
D0
D11
D21
M
+
IDO1
Granulocytes
GAPDH
IDO1
Monocytes
GAPDH
B
Pt#1
Pt#2
C
Day of treatment (0, 7, 11, 21)

Supplement: Additional file 1 — Expression of IDO1 mRNA and serum Kyn levels in patients given pegfilgrastim. Panel A: Expression of IDO1 mRNA in patient monocytes and granulocytes. Details on RNA extraction and reverse-transcription were previously published [8]. The following primers were used for mRNA amplification: 5'-ACTGCCCCTGTGATAAACTGTGG-3' and 5'-GCGTGTGCCATTCTTGTAGTCTG-3' (human IDO1; GI 156071492); 5'-TGACATCAAGAAGGTGGTGA-3' and 5'-TCCACCACCCTGTTGCTGTA-3' (human GAPDH; GI 7669491). Primer sets were designed using the Beacon Design Software (Version 3) and the sequences available in the Gene Bank™ database. All nucleotide primers were synthesized by MWG (Florence, Italy), and PCR products were analyzed on 3% agarose gel (Agarose, type XII: low viscosity for beading, Sigma Aldrich) stained with ethidium bromide. M = marker. + = normal endometrial tissue used as positive control for IDO1 mRNA expression. Panel B: Quantitative densitometry (Quantity One software; Bio-Rad, Hercules, CA) is shown with monocytes and granulocytes isolated from 2 patients given pegfilgrastim. Insufficient numbers of cells were available on day +7, and PCR analyses were performed with patient material obtained on days 0, +11 and +21. Normal endometrial tissue was used as positive control for IDO1 mRNA expression (red column). Panel C: Serum Kyn levels were measured by RP-HPLC in 5 patients before (day 0) and after pegfilgrastim administration (days +7, +11 and +21), as detailed in Materials and Methods. Data from each individual patient have been plotted using a different color. The dotted line indicates the median serum Kyn concentration measured in 50 healthy subjects (2.3 μM). [file 1479-5876-8-114-S1.PPT]

## Slide 1
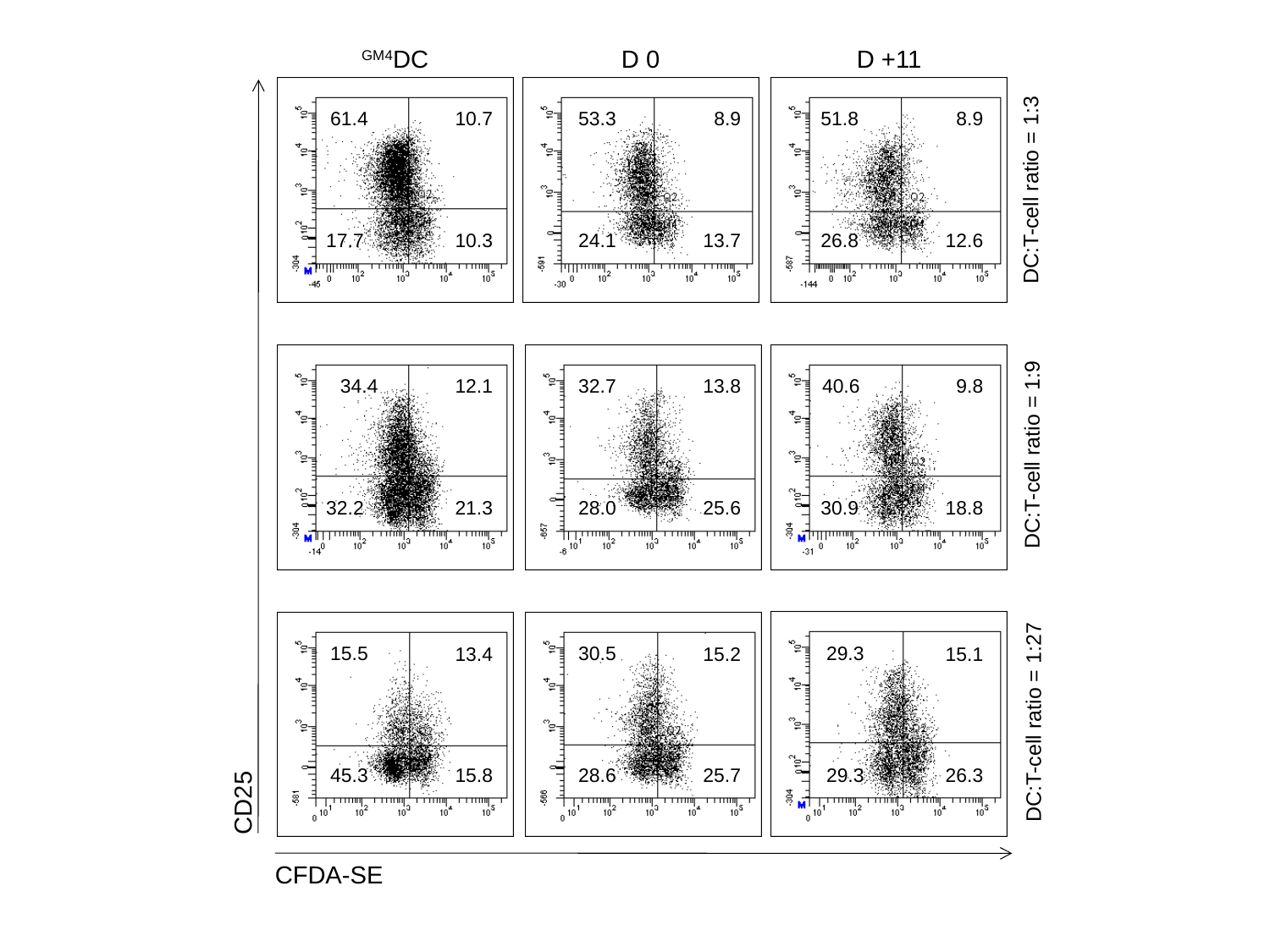

GM4DC
D 0
D +11
61.4
10.7
53.3
8.9
51.8
8.9
DC:T-cell ratio = 1:3
17.7
10.3
24.1
13.7
26.8
12.6
34.4
12.1
32.7
13.8
40.6
9.8
DC:T-cell ratio = 1:9
32.2
21.3
28.0
25.6
30.9
18.8
15.5
30.5
29.3
13.4
15.2
15.1
DC:T-cell ratio = 1:27
45.3
15.8
28.6
25.7
29.3
26.3
CD25
CFDA-SE

Supplement: Additional file 2 — T-cell stimulation by Mo-DC generated in vitro after in vivo administration of pegfilgrastim. Mo-DC were differentiated from patient monocytes in the presence of either pre-G-CSF serum or post-G-CSF serum (collected on day + 11), as detailed in Materials and Methods. Immunogenic DC were generated with IL-4 and GM-CSF, in accordance with established DC differentiation protocols [17]. The Mo-DC preparations were co-cultured with CFDA-SE pre-loaded, allogeneic naïve CD4+CD25- T cells at different T cell-to-DC ratio. The percentage of CD25-expressing, CFDA-SEdim and CFDA-SEbright cells is indicated. One representative experiment out of 5 with similar results is shown. [file 1479-5876-8-114-S2.PPT]

## Slide 1
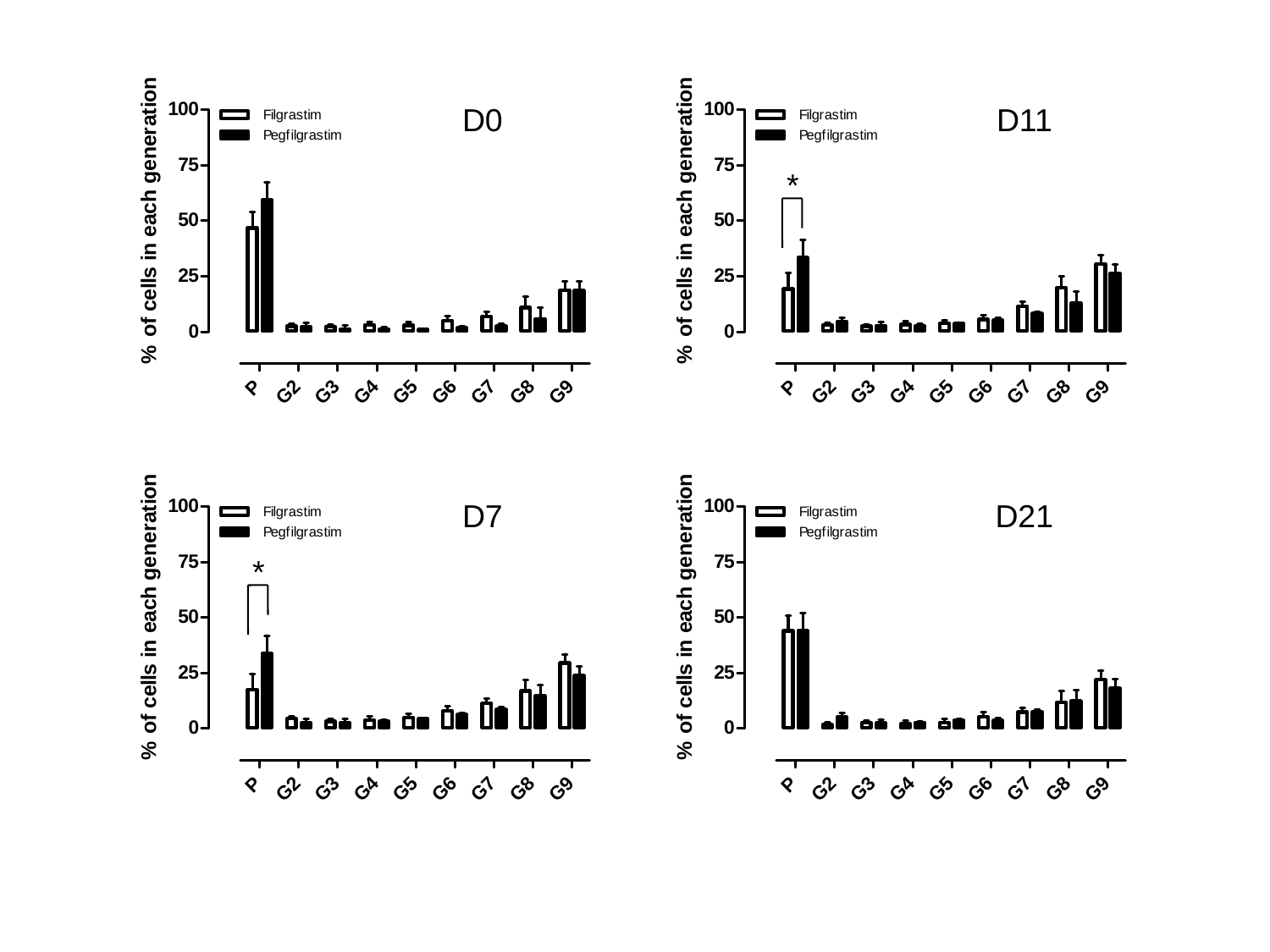

*
D0
D11
D7
D21
*

Supplement: Additional file 3 — Cell proliferation tracking after provision of post-G-CSF serum to MLR cultures. MLR cultures were established as above detailed. T cells and monocytes were plated at a fixed DC-to-T cell ratio (1:27). The percentage of proliferating T cells residing within each cell generation (G) was calculated with the proliferation wizard of the ModFit™ software. Median values and interquartile range are shown. *denotes a p value < 0.05 when comparing the percentage of parental (P), undivided cells in MLR cultures established with serum from patients given pegfilgrastim (black bars) or filgrastim (empty bars). [file 1479-5876-8-114-S3.PPT]
